# Preconception Care: A New Standard of Care within Maternal Health Services

**DOI:** 10.1155/2016/6150976

**Published:** 2016-05-29

**Authors:** Stephen J. Genuis, Rebecca A. Genuis

**Affiliations:** ^1^Department of Obstetrics and Gynecology, University of Alberta, Edmonton, AB, Canada T5H 3V9; ^2^University of Calgary, Calgary, AB, Canada; ^3^Millcreek Environmental Health Clinic, Edmonton, AB, Canada T6K 4C1

## Abstract

Emerging research suggests that much pediatric affliction has origins in the vulnerable phase of fetal development. Prenatal factors including deficiency of various nutrients and exposure to assorted toxicants are major etiological determinants of myriad obstetrical complications, pediatric chronic diseases, and perhaps some genetic mutations. With recent recognition that modifiable environmental determinants, rather than genetic predestination, are the etiological source of most chronic illness, modification of environmental factors prior to conception offers the possibility of precluding various mental and physical health conditions. Environmental and lifestyle modification through informed patient choice is possible but evidence confirms that, with little to no training in clinical nutrition, toxicology, or environmental exposures, most clinicians are ill-equipped to counsel patients about this important area. With the totality of available scientific evidence that now exists on the potential to modify disease-causing gestational determinants, failure to take necessary precautionary action may render members of the medical community collectively and individually culpable for preventable illness in children. We advocate for environmental health education of maternity health professionals and the widespread adoption and implementation of preconception care. This will necessitate the translation of emerging knowledge from recent research literature, to health professionals, to reproductive-aged women, and to society at large.

## 1. Introduction

The twentieth and twenty-first centuries have witnessed remarkable advances in maternal-fetal medicine. Puerperal fever causing maternal death, once a plague threatening the life of each and every woman entering a maternity ward [1], now rarely occurs in developed nations with modern obstetrical care [2, 3]. Moreover, neonatal and infant death rates over the last century have fallen precipitously in most developed countries [2, 3]. Juxtaposed with such epic advancement in obstetric and pediatric healthcare, however, we now face different and emerging concerns in the multifaceted area of maternal-fetal medicine.

Over the last few decades, there has been a significant rise in the incidence of preterm birth, a problem often associated with short- and long-term health issues for offspring [4, 5]. The Institute of Medicine estimated the annual costs for the burden of morbidity, disability, and mortality associated with preterm birth in the United States to be at least $26.2 billion [6]. With potential impairment of optimal biome development [7], Caesarean delivery has nevertheless become increasingly common with rates in America soaring from 5 percent in 1970 to 32.7 percent in 2013 [6, 8]. The prevalence of mental health problems has rapidly escalated throughout the world [9] with postpartum depressive illness continuing to ravage the well-being of countless young mothers [10]. Finally, the pandemic of diabetes, including gestational glucose intolerance, affects increasing numbers of reproductive aged women [11].

*Rise in Pediatric Chronic Illness*. In addition to various challenges in the field of maternal health, the marked rise in chronic and developmental illness in offspring [12] has been an issue of particular concern in the medical community. With chronic disease overtaking infectious disease as the major burden of pediatric affliction [13], rates of a broad spectrum of enduring childhood conditions have been climbing sharply and, at the present time, show no signs of changing direction [12]. Furthermore, it is becoming increasingly evident that modifiable prenatal factors may be significant determinants in many of these health problems, a reality which recently prompted FIGO (the International Federation for Obstetrics and Gynecology), an organization which represents obstetrical and gynecological associations from 125 countries, to release a special communication urgently calling for concerted action in the field of maternity healthcare as it relates to preventable childhood disorders [14].

Rates of autism, for example, have risen steeply and emerging evidence suggests that this rise, in part, may be attributed to preventable gestational determinants [15]. Other neurodevelopmental disorders, learning disabilities, and impaired IQ in children have also been associated in some cases with modifiable prenatal factors [16]. Asthma, another condition potentially linked to prenatal determinants [17], involved about 5% of children in 1965 yet now affects over 20% of children in some areas [18]. Furthermore, there are many recent publications attributing the swelling pandemic of pediatric allergy to modifiable prenatal factors [19–25]. Some chromosomal abnormalities [26] as well as various congenital anomalies including open neural tube defects [27] and certain cardiac abnormalities [28] have been linked in some cases to modifiable gestational factors. Hypospadias, an anomaly linked in some cases to prenatal determinants [29], previously affected 1/500 newborn baby boys and now is found in 1/125 male infants [30]. Furthermore, many lethal pediatric and early adolescent cancers have recently been directly attributed, in large part, to prenatal determinants [31]. Emerging research, however, suggests that the impact of gestational determinants on offspring does not end with childhood.

Increasing recognition of fetal origins of adult disease [32] continues to unfold as various prenatal determinants have been associated with adolescent and adult-onset disease including multiple sclerosis [33], eating disorders [34], cardiovascular disease [35], various metabolic disorders [36], and some cases of compromised bone health [34]. In review, it is becoming increasingly apparent that gestational determinants may exert a significant short- and long-term impact on health and well-being. The question arises: if modifiable gestational factors are directly responsible for various obstetric complications and myriad pediatric health problems, is it possible to modify such determinants in order to prevent the development of such morbidity and mortality? This publication will endeavor to (i) explore the etiology of the recent rise in pediatric and maternal-fetal health complications, (ii) discuss the high cost of adverse gestational outcomes, and (iii) advance the case for preconception care as a new standard of care within the spectrum of maternal health services.

## 2. A Paradigm Shift in the Understanding of Disease Etiology

We are on the cusp of change in our scientific understanding of disease etiology. Throughout human history, scientists and philosophers have sought to uncover the underlying cause of illness and suffering [37]. With wide swings in disease attribution from the metaphysical (the predominant belief when Hippocrates, the Father of Western Scientific Medicine, came onto the scene circa 400 BC [38]) to the germ theory of the late 19th century, conventional wisdom in much of the 20th and early 21st century has attributed most chronic and otherwise inexplicable illness to genetic factors and the “bad luck” hypothesis. With the recent surge of epigenetic research in the last two decades, however, the belief that those with chronic illness are ill-fated victims of cosmic genetic roulette is quickly fading [39].

**Figure 1:**
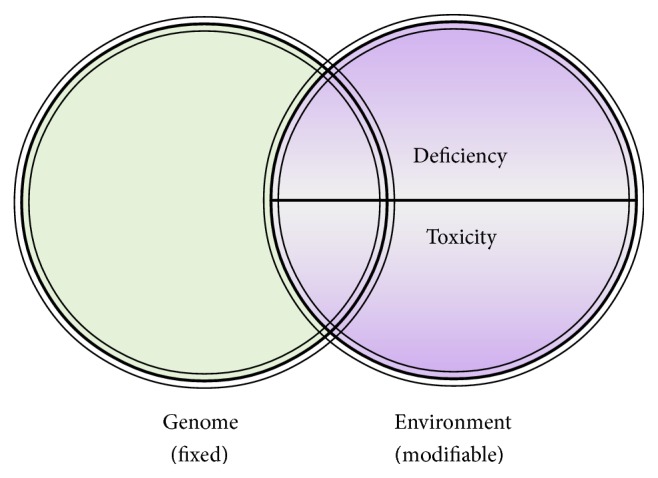
Etiology of illness.

With marked inconsistency of outcomes in identical twin studies [40, 41], with the changing profile of disease incidence and prevalence in geographic pockets [42, 43], with shifting health outcomes associated with migration [44], and with extensive research into molecular determinants of illness [39], it is becoming abundantly apparent that virtually all disease, including affliction in the gestational period, is the result of the interaction between our genes and the environment (Figure 1) [45, 46]. In fact, recent evidence confirms that modifiable environmental factors appear to be responsible for 70–90 percent of illness [47, 48]. In other words, changeable determinants within our environment are interacting with our genome to maintain health or cause illness [39].

Yet, within the environmental domain, there appear to be only two determinants which make up the environment sphere: (i) are we getting what we need and (ii) are we being exposed to things that are toxic [45]? Simply put, for any functional system including the human organism to develop and thrive, it must receive determinants which are required and avoid those which are harmful [45]. During gestation in particular, it appears that the exquisitely intricate processes that direct the growth and development of early human life are profoundly sensitive to nutritional requirements and vulnerable to environmental insults. Insufficiency of required nutrients or minute adverse exposures during critical phases of development, for example, may have serious and life-long consequences [49].

Furthermore, it is often thought that various chromosomal abnormalities are simply caused by random, arbitrary genetic mutations. Rather than attributing such outcomes exclusively to our genes, however, recent evidence suggests that this environmental model may also apply to some chromosomal abnormalities. Deficiencies of certain nutrients, for example, are associated with higher rates of chromosomal abnormalities including Down's syndrome [26], and low dose chemical exposures have been found to induce genome instability with enhanced tendency for novel genetic mutation [50].

A further point of particular importance with regard to the gestational phase in the continuum of life is the pronounced vulnerability of the developing child in utero, as a result of marked physiological differences between what is found in a developing fetus and the inner workings of a toddler or an adult. For example, there is distinct susceptibility to harmful agents during gestation, and exposure levels that may not appear to harm pregnant women, such as with alcohol exposure, may have a profound impact on the fetus [51–54].

A number of physiological factors within the in utero environment account for this exquisite fetal vulnerability to exposures which include the following:

- The placental unit is unable to filter the contemporary array of chemical toxicants.
- The fetal liver is immature and incapable of efficient detoxification of contaminants.
- Low levels of fetal binding proteins result in high unbound fractions of bioactive toxicants.
- Excretion pathways are undeveloped and excreted urinary pollutants are recycled with repeated reuptake into the nose and mouth through the amniotic fluid.
- The blood-brain barrier is immature and more permeable to adverse chemical agents.
- Compared to mother, there is higher toxicant concentrations by weight in the fetus [49] which then marinate rapidly developing fetal organs.

These factors all mark the prenatal period as a time of unique propensity for untoward effects [55] and explain why adverse agents tolerated by the mother may damage the rapidly growing tissues of her child [56]. Such factors may also explain why levels of some toxic agents, such as the teratogenic toxic element mercury, accumulate in fetal tissues with concentrations considerably higher in offspring than in fish-consuming mothers [57].

Furthermore, there is absolute requirement at critical stages of development for essential nutrients in order to secure proper formation, differentiation, and development of fetal tissues. While toddlers and adults may tolerate a temporary insufficiency of specific nutrients and recover when nutritional requirements are replete, the developing child in utero may have altered development and enduring anomaly when essential nutritional biochemicals are lacking at critical periods; it is well-recognized with folate deficiency in early gestation, for example, that insufficiency of required nutrients can have profound long-term implications [26, 58].

In review, it appears that most human illness, including pregnancy-related disease, is etiologically related to deficiency and toxicity (Figure 1) [45, 46]. Accordingly, the essence of producing a healthy child and avoiding the short- and long-term problems associated with fetal and obstetrical complications is to secure adequacy and avoid toxicity in the gestational phase; this is the foundation and objective of good preconception and prenatal care. As such, it is worth considering what every woman should be adequately apprised of prior to and during her gestation in order to secure optimal health for herself and her child.

## 3. Requirements for a Healthy Pregnancy and Infant

Since its inception, the constitution of the World Health Organization (WHO) defined health as a state of complete physical, mental, and social well-being, and not merely the absence of disease or infirmity [59]. For the purposes of this paper, however, we will confine ourselves to discussion of physical requirements for optimal health during the gestational period. Broadly speaking, the human organism needs many fundamental determinants in order to thrive: clean air, fluids, nutrients, rest, sunlight, exercise, healthy microbiota, and so on. Recent research suggests that gestational nutritional deficiencies and insufficiency of optimal biome development in the neonate remain ubiquitous modifiable factors that are contributing to widespread maternal and/or pediatric compromise.

**Figure 2:**
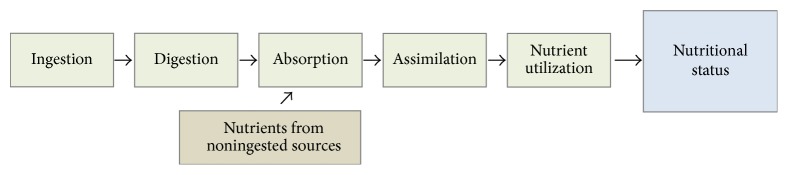
Determinants of nutritional status.

Some health professionals believe that nutrient insufficiency is only a problem among indigent and disadvantaged populations. Many feel that women in the developed world who eat regularly are getting all they need for themselves and their developing children from their diet. These perspectives can be misguided, however, for a number of reasons that have recently come to light including the following:

- There has been a major transition in the way that people eat over the last few decades [60].
- There has been a significant decline in the nutritional content of some foods [61].
- Various processes such as genetic modification of crops and multipesticide application may modify the overall biochemical composition of some common food staples [62].
- Many important nutrients come from sources other than foods, such as human microbiota and sunlight [63].
- Nutrient intake does not automatically translate into nutritional status (Figure 2). Various acquired metabolic errors resulting from factors such as toxicant exposures can interfere with digestion, absorption, assimilation, or utilization of nutrients [64–66].

A woman's nutritional status, however, must be adequate in order for her to support a healthy pregnancy. Various nutritional deficiencies in pregnancy can have dramatic effects on the incidence of illness in her offspring. As mentioned, folate deficiency is perhaps the most well-known example, as without sufficiency of this critical B vitamin, the risks of open neural tube defects [67], miscarriage [58], and Down's syndrome [26] are significantly elevated. Of late, however, several other micronutrient deficiencies have recently come to light: (a) gestational iron deficiency, for example, is associated with cognitive and immune impairment in offspring [68]; (b) maternal iodine requirements rise by 50% in pregnancy to meet fetal requirements; insufficiency can lead to pediatric hypothyroidism and intellectual disabilities [69]; (c) concerns have emerged about potential fetal sequelae, including cleft palate, of maternal biotin deficiency [70]; and (d) there appears to be increased risk for neural tube defects with low maternal vitamin B12 status [27]. With the recognition of these common gestational deficiencies, great public health efforts have been made to support nutritional adequacy by population-wide fortification of various foods and routine gestational supplementation with a vitamin/mineral supplement.

Nonetheless, two issues arise with this approach. As will be discussed, it has recently been found that some common prenatal supplements used to preclude deficiency are contaminated with toxicants [71], thus providing a daily dose of pollutant to the developing child. Accordingly, caution must be taken when considering the source of supplements taken during gestation. Furthermore, rather than relying on supplementation, healthy foods should be the source of most nutrients. Recognizing the widespread problem of fetal prepollution with assorted chemical toxicants [14, 49, 72] and the reality that foods are an increasing source of toxicant exposure [62], the authors recommend a diet with the maximum amount of nutrition and the least amount of toxicant contamination (a credible organic food diet) to supply most required nutrients [73]. Secondly, despite healthy eating, there are various micronutrient deficiencies of critical importance which are often overlooked in the care of pregnant women. We will briefly mention three common micronutrient deficiencies.

### 3.1. Vitamin D

There is general consensus in the medical literature that the vitamin D status of many individuals and population groups throughout much of the world (as reflected by population measurements of serum 25(OH)D levels) is insufficient for optimal health [74]. Pregnant women screened in one study in Western Canada, for example, demonstrated that the majority of women in one city had clearly inadequate vitamin D levels [75]. While a healthy 25(OH)D level throughout pregnancy is considered to be in the 100–150 nmol/L range [76], 76% of pregnant women in this study had levels under 80 nmol/L and 23% had levels under 40 nmol/L [75]. Recent evidence, nonetheless, suggests that this critical nutrient is involved in the regulation and expression of over 2700 different genes [77]. Recognition of the burden of health sequelae resulting from low maternal vitamin D concentrations continues to expand (Tables 1 and 2).

The literature has discussed that vitamin D insufficiency may be associated with first trimester pregnancy loss [85, 86], gestational diabetes [103, 104], preeclampsia [89, 105], preterm birth [92, 93], higher rates of primary and emergency Caesarean delivery [94, 106], small for gestational age infants [95], and maternal postpartum depression [96]. The long-term resources required to address the consequences of these conditions are enormous. Furthermore, adverse gestational complications do not end with delivery. It is well-recognized that children born preterm, for example, have much higher rates of chronic physical and mental health problems [4, 107]. The estimated 10-year costs to care for children born preterm in Canada and the United States, for example, is staggering [6, 108].

An interesting cohort study correlating maternal vitamin D deficiency at 18 weeks' pregnancy and health outcomes of progeny found that gestational vitamin D deficiency is associated with impaired lung development in 6-year-old offspring, neurocognitive difficulties at age 10, increased risk of eating disorders in adolescence, and lower peak bone mass at 20 years [34]. In addition, gestational vitamin D levels may even impact adult health as there is early evidence that vitamin D sufficiency in pregnancy may have a protective role in the development of adult-onset multiple sclerosis [33]. With abundant evidence of myriad health sequelae associated with gestational vitamin D deficiency, there is potential for considerable amelioration of maternal and fetal health outcomes by educational and healthcare measures in the preconception and prenatal period to secure vitamin D sufficiency throughout gestation [76]. Because of widespread vitamin D insufficiency in many population groups and inconsistent response to fixed doses of vitamin D supplementation, the authors recommend testing for 25(OH)D levels and suitable intervention as required [76].

### 3.2. Magnesium

Another micronutrient which is commonly deficient in much of the general population is the essential mineral magnesium [109]. This important nutrient is a cofactor in more than 300 enzymatic reactions required for the proper function of proteins, mitochondria, and nucleic acids [110]. Yet, it is estimated that about half of American adults do not receive the average daily requirement for magnesium [109, 111]. Furthermore, it is difficult to diagnose magnesium deficiency as total body levels are difficult to assess given its primarily intracellular and intraosseous nature. In other words, serum testing is inadequate to determine the magnesium status of the body as only about 1% of magnesium is found in plasma and red blood cells [110, 112].

Widespread magnesium insufficiency is occurring for a number of reasons including the following:

- Reduced dietary intake of foods replete in magnesium is widespread [109]. Common staples such as meat, dairy, sugar, and white flour have little to no magnesium.
- Cooking and boiling of produce results in a significant decline of the food's magnesium content [113].
- There are diminished levels of magnesium in many processed foods and some nonorganic foods [114]. Most foods in grocery stores are processed.
- Reduced gastrointestinal absorption of magnesium occurs in the face of vitamin D deficiency. As discussed, most pregnant women appear to be deficient in vitamin D.
- Medications in common usage (e.g., some antibiotics, antacids, and hypertensive drugs) diminish absorption of magnesium.
- Some commonly used pesticides have the propensity to chelate minerals [115], decreasing the content of magnesium in soil and some crops [116].
- There is excess excretion of magnesium with alcohol use and the presence of type 1 or type 2 diabetes [110].
- There is an increased requirement for magnesium in pregnancy [117].
- Evidence demonstrates increasing soil depletion of certain essential nutrients as a result of fertilization techniques not providing the spectrum of required minerals [61].
- There has been the expansion of monoculture agricultural techniques which have a tendency to consume and deplete specific nutrients.

The potential health sequelae of insufficient magnesium intake in the general population are numerous including asthma [118], cardiovascular disease [119], mental health problems [120], bone health compromise [121], and development of some cancers [122]. Although magnesium deficiency occurs frequently in pregnancy [123], the consequences of gestational magnesium deficiency are only beginning to be studied. Preliminary evidence suggests that maternal magnesium sufficiency may be linked to pregnancy outcomes as well as long-term health of the infant [80]. For example, oral magnesium supplementation given before the 25th week of gestation compared with placebo was associated with a lower frequency of preterm births, low birth weight infants, and fewer small for gestational newborns [124]. In one study, magnesium supplementation in pregnancy was associated with lower mean arterial pressure in women along with higher birth weight infants and fewer days spent by offspring in the neonatal intensive care unit [81]. There is also early evidence that fetal hypomagnesemia may be associated with metabolic syndrome later in life [123].

At this point, however, the consensus is that there is insufficient high quality evidence to conclude that dietary magnesium supplementation should be routinely recommended in pregnancy [125]. If concern about magnesium adequacy is present at assessment of individual patients, the authors suggest that a cautious approach may be to supplement women in the preconception period and then educate them about dietary sources to acquire sufficiency throughout their pregnancy.

### 3.3. Docosahexaenoic Acid

Docosahexaenoic acid (DHA) is an essential omega-3 fatty acid found most abundantly in seafood. As conversion to DHA from other primary omega-3 fatty acids is variable and dependent on enzymatic availability as well as functionality of metabolic conversion mechanisms, DHA requirements generally focus on direct ingestion of seafood. With increasing awareness of widespread environmental contamination of seafood by assorted toxicants including mercury, however, more and more women are avoiding consumption of fish and other marine products during pregnancy [79].

If not addressed, the ensuing gestational DHA insufficiency may be problematic as inadequate DHA has been linked to numerous deleterious health outcomes for both mother and child. DHA deficiency has been associated with preterm labour [126], pregnancy induced hypertension [127], and postpartum depression in women [128]. In addition, children born to women with inadequate DHA may be at an increased risk for central nervous system disturbances [129], poor sleep patterns [130], lower IQs [131], and impaired blood pressure control later in life [132]. Education through preconception care about the need and means to secure gestational DHA requirements [133, 134] can significantly reduce the risk for a constellation of maternal and fetal adverse health outcomes associated with DHA insufficiency.

### 3.4. Healthy Biome

A healthy human biome, the biological ecosystem of living organisms found within and upon each person, appears to be a critical and often unrecognized requirement of the human organism [135]. Within this biome, sometimes referred to as the last human organ [136], there are approximately ten times more microbial cells than human cells due to the abundance and diversity of the naturally occurring microbiome [135]. There is a plethora of ongoing research currently underway to further understand the biome and its practical application to health and disease. As the NIH (National Institutes of Health) sponsored Human Microbiome Project [135] is still in its early stages, however, there remains much uncertainty about the microbiome of the in utero environment and its relation to maternal-fetal health outcomes.

It was traditionally thought, for example, that the womb environment was sterile; this idea is being challenged, however, as studies examine the germ environment of the placenta, meconium, and the newborn [137–140]. What is certain, however, is that the maternal flora and infant's journey amidst the vaginal flora of the birth canal are important determinants of gestational and pediatric outcomes [63]. Dysbiosis, a disordered germ ecosystem, may place a pregnant woman, for example, at increased risk for preterm labour [82]. Bacterial vaginosis is a manifestation of an altered vaginal biome where there is a dramatic reduction in lactobacilli and increase in anaerobes, a finding that has been association with early onset of labour [141–143].

It appears that the maternal microbial flora changes significantly throughout pregnancy [144] and is decidedly distinct from that of a nonpregnant female. This transition is thought to be important for the developing fetus [145] and may be involved in normal fetal growth and development. There is concern, however, that alteration of the maternal microbiome at any stage during pregnancy may have an impact on the developing child and gestational health. It is becoming evident, for example, that antibiotic use during pregnancy may potentially disrupt normal maternal-offspring microbiota exchange. New evidence regarding maternal gut-fetal brain connections in animals suggests that use of certain antibiotics around the time of conception may be associated with antisocial behavior and anxiety in offspring [146] and recent human work has linked a higher offspring risk of childhood obesity with maternal use of antibiotics in the second or third trimester [84].

Moreover, if a woman tests positive for vaginal Group B* Streptococcus* (GBS) towards the end of her pregnancy (as is found is a significant portion of the normal healthy population) she is often administered antibiotics throughout her labour potentially leading to changes in her flora and an altered gastrointestinal microbiome in her neonate. As a result, it appears that such offspring are subsequently more susceptible to serious bacterial infections in their first three months of life [83, 147]. Gestational antibiotic use is only one factor that may affect the biome: other factors include unfiltered chlorine in drinking water [148] and exposure to certain pesticides in food or drink [149].

Pediatric outcomes associated with mode of delivery also appear to verify that the maternal urogenital tract microbiome plays an important role in pediatric health and disease [150]. The link between mode of delivery and subsequent childhood pathology is important with evidence of microbial colonization differences between children born vaginally and those born by Caesarean delivery [7, 151, 152]. It appears that such differences in the constitution of the biome are significant in relation to subsequent health. In fact, Pediatrics, the official journal of the* American Academy of Pediatrics*, recently released a paper which concluded that “children delivered by Caesarean delivery had significantly increased risk of asthma, systemic connective tissue disorders, juvenile arthritis, inflammatory bowel disease, immune deficiencies and leukemia” [153]. Other studies have reported higher rates of celiac disease [154], type 1 diabetes [155], and allergic disease [152, 154] among those born by Caesarean delivery.

It is increasingly evident that transmission of a healthy microbiome to the neonate during vaginal delivery may affect health from birth to adulthood [156–158]. As operative delivery via Caesarean section may result in deficiency or disruption of a healthy biome, steps to diminish the need for operative delivery by addressing potentially modifiable determinants such as elevated BMI [159] and vitamin D deficiency [94] in pregnancy may be in order. Knowledge about the fetal biome may also influence decisions regarding trials of vaginal birth after previous operative deliveries. For example, it is evident that infants delivered after a long labour with a dilated cervix may be exposed to a different microbial environment compared to a child born by elective Caesarean delivery. The risk of asthma, for example, was increased considerably in female offspring of women who underwent a repeat Caesarean delivery without ruptured membranes versus those born in situations with ruptured membranes and/or labour prior to Caesarean delivery [160].

Furthermore, there is increasing discussion of a novel technique entitled “vaginal seeding” [161], using gauze to gather a mother's birth-canal bacteria and then applying them to the Caesarean delivered infant's mouth, skin, and eyes, which results in the baby's biome more closely resembling that of vaginally born babies [161]. At this point, it is unclear whether the routine use of probiotics or fermented foods as a source of healthy organisms has any role in preconception or prenatal care. As we continue to learn more about the important role of the maternal and fetal biome, it seems beneficial to educate women about the need to avoid interventions or exposures whenever possible that may be detrimental to their and their child's germ ecosystem.

## 4. What Ought to Be Avoided for a Healthy Pregnancy and Infant

It is widely recognized that maternal exposure to adverse chemicals in cigarette smoke, teratogenic medications, or illicit drugs during pregnancy can have adverse consequences for gestational outcomes and the developing fetus. According to the American Academy of Pediatrics, maternal exposure to any amount of alcohol is now considered unsafe throughout pregnancy because of the potential serious and enduring impact that this chemical might have on a developing child [54]. In each of these situations, it has been appreciated that fetal exposure to potentially toxic substances can be a determinant of chronic adverse sequelae. In the last half century, however, there has been a dramatic escalation of adverse gestational exposures as a result of (i) the chemical revolution, with the introduction and release of tens of thousands of anthropogenic chemicals untested for impact on human health, and (ii) the expanding electrical revolution, with the near ubiquitous exposure to electromagnetic radiation from wireless communication devices, power lines, and myriad other sources. In this section, we will explore some of the emerging evidence of gestational exposures from these two sources.

### 4.1. Chemical Exposures

Over the last few decades, we have saturated the environment with an exceptional number of untested, unsafe, and unregulated chemical compounds; we are just beginning to understand the consequences of what we have done. The release of thousands of chemical agents into our air, water, soil, and foodstuffs has now resulted in everyday exposure for most people to adverse toxicants primarily through foods and fluids consumed, personal care products applied to skin, and especially through the inhalation of polluted air. With mounting evidence of the particular danger of exposure to toxic chemicals during the gestation period, FIGO (the International Federation for Obstetrics and Gynecology) released a special communication at their Vancouver convention in the fall of 2015, with the explicit message that “exposure to toxic environmental chemicals during pregnancy and breastfeeding is ubiquitous and is a threat to healthy human reproduction” [14].

Although the sequelae of toxicant contamination include a wide range of health problems from autism [15] to mental illness [162], to cancer [31], and to widespread morbidity and mortality from indoor air pollution [163], the in utero phase of the life cycle is a time of particular vulnerability to toxic chemical exposure from assorted sources. The developing fetus in the seemingly isolated world within the amniotic sac has unique physiological characteristics, as previously discussed, that predispose to significant harm once exposure is introduced. As a result, recent cord blood research confirms that most infants are being prepolluted even before their very first breath and that chemical exposure with fetal bioaccumulation is increasingly a routine phenomenon rather than a sporadic event in modern society [14, 164, 165].

While some suggest that the risk of toxic exposure is irrelevant because toxicant levels are very low, the National Academy of Sciences has concluded that, like gestational alcohol exposure, any level of exposure to toxic chemicals should be assumed to be potentially harmful, that is, that there is no “safe dose” [166]. This important point is punctuated by considering the reality of normal biochemistry. For example, serum estradiol levels as minuscule as 30 parts per trillion in some phases of the menstrual cycle [167] are enough to regulate and control various physiological functions in adult women; exposure of tiny developing infants to hormonally active endocrine disrupting toxic chemicals at serum levels hundreds and thousands of times higher in parts per billion and parts per million may have potential adverse biological impact [168]. The recent FIGO special communication thus concludes that “there are tens of thousands of chemicals in global commerce, and even small exposures to toxic chemicals during pregnancy can trigger adverse health consequences” [14].

**Figure 3:**
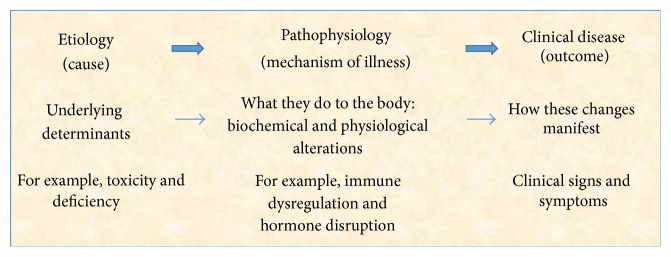
Pathway to clinical disease [45].

Observational studies of the impact of such contamination on subsequent health and well-being are the subject of much contemporary research and the emerging findings are sobering indeed (Table 3). Toxic chemicals can impact human metabolism in many ways including hormone disruption [198], epigenetic alteration [199], immune dysregulation [200], direct cytotoxicity [201], carcinogenesis [31], mitochondrial impairment [202], and oxidative damage [203]. The clinical consequences of such pathophysiological disturbances include endocrine disorders, assorted cancers, congenital anomalies, autoimmune disease, allergic states, pediatric and adult neurological conditions, reproductive failure, psychiatric disability, and many other disease states (Table 3). Furthermore, recent evidence suggests that some of the toxicant-induced metabolic alterations can induce epigenetic change and transmit for multiple generations [204, 205]. In review, the evidence clearly demonstrates that numerous toxicants are etiologically igniting assorted pathophysiological mechanisms which are consequently resulting in clinical disease states (Figure 3).

It was once assumed that the placental barrier acted to safeguard a growing child from any dangerous exposure a mother might have. Over time this has been demonstrably shown to be false as medications, alcohol, and other exposures have had obvious effects on children, leaving no doubt that maternal exposure to toxicants results in contamination of the developing child. The extent to which this is true, however, has become frighteningly clear in the last 15 years as studies of umbilical cord blood have been carried out. In 2004, a sample of American neonates had their cord blood tested for a limited variety of different chemicals. On average, nonetheless, each individual child's sample was found to contain 200 unique toxicants, including pesticides, heavy metals, and many other pollutants [72]. A similar Canadian analysis of three newborns found 137 different compounds in their cord blood, with each child's sample containing between 55 and 121 identified toxicants [165].

One of the more significant findings in these studies was that toxic chemicals banned several decades ago such as polychlorinated biphenyls (PCBs) were detected in the cord blood of children born recently. These findings confirm that bioaccumulated toxicants in mothers from long ago, not only exposures occurring during the current pregnancy, pose a significant risk for the developing child. These findings are concerning, given that many detected chemicals are known to be pathophysiologically active within human tissues. The implications of such scientific research are clear: in addition to precluding exposure to the constellation of toxic agents during each gestation, the importance of detecting accrued compounds and clearing them from the mother's body [206–208] prior to pregnancy is apparent. A brief review of some of the more common exposures follows: details on testing to assess for toxicant burdens and techniques to facilitate elimination of such toxicants are beyond the scope of this work but are found in other publications [206–211].

#### 4.1.1. Household and Vehicle Exposures

It is acknowledged that inhalation of contaminants in the air is the most common source of toxicant accrual and explains why, according to the World Health Organization, mortality related to air pollutants, primarily within the home, accounts for about 8 million deaths annually [163]. While more and more people are understandably concerned about the quality and purity of their food, drink, and personal care products they often pay less attention to contaminated air, the largest single environmental health risk [163], and the major source of toxic chemical exposure for most reproductive age women. To highlight this point, one might consider the following: while many individuals in the western world apply about 10–20 mls of personal care products to their skin daily and eat and drink up to about 3 litres per day, each adult breathes around 10,000 litres of air per day. Accordingly, exposures within the indoor air environment of the home, car, and workplace are something that needs to be carefully considered, especially by those planning a pregnancy.

Within the home there are myriad common sources of airborne exposure and contamination, including flame retardants off-gassing from furniture and mattresses, assorted chemicals released from personal care products such as hair sprays, myriad toxicants emitted from the vents of electronic equipment like computer printers, nonstick compounds discharged from synthetic carpeting, solvents released from petroleum-based candles, formaldehyde off-gassing from wood glues and certain wood products, chloroform gas produced from showers using unfiltered chlorinated water, and on and on (see Table 3). Toxic exposures, often from airborne sources during pregnancy, have been associated with myriad adverse outcomes like pediatric allergy [21], decreased IQ [169], infectious disease [25], pediatric endocrine disorders [179], respiratory illness [174], autism [15], childhood cancers [31], increased hyperactivity scores [169], congenital birth defects [28], and various other childhood health problems. Once patients are sufficiently educated, however, simple measures such as air purification techniques can often be instituted to eliminate, or at least minimize, exposure and thus preclude consequent sequelae of toxicant-induced metabolic alteration.

Toxicant exposure within vehicles remains poorly studied but represents a chief source of air pollution for many women in the western world. With a small volume of air inside the cabin of most vehicles and the disproportionately high concentration of airborne contaminants delivered to this confined space originating from (i) surrounding traffic exhaust containing myriad toxic pollutants [212, 213], (ii) emissions off-gassing from assorted metal, plastic, and petroleum components from a heated engine, and (iii) discharges from upholstery, air fresheners, plastic components, flame retardants, and so on within the cabin, vehicle inhabitants are continuously inhaling untold concentrations of airborne contaminants [213]. As many women, including those who are pregnant, spend more than one hour per day in their cars, respiring about 400–500 litres of contaminated air each hour, credible air filtration and purification within the car is extremely effective in diminishing toxic exposure to the mother and developing child.

#### 4.1.2. Maternal Occupational Exposures

There is extensive evidence in the literature that maternal occupational exposure to adverse chemicals in a variety of jobs and professions is directly linked to adverse outcomes in children [28, 214–217], a fact that is often ignored in health discussions with pregnant women. For example, childhood leukemia has been strongly associated with maternal exposure to solvents, paints, and petroleum products during pregnancy as what might occur in a variety of occupations [218]. In fact, women working with solvents in pregnancy have a dramatically higher rate of children born with cardiac and neurological abnormalities [28] and their offspring are also more likely to obtain lower scores on intellectual, language, motor, and neurobehavioral functioning [217].

Occupations involving maternal exposure to traffic-related air pollution may place offspring at increased risk for adverse outcomes including cardiac defects [219, 220] while maternal work as a janitor or maid, where airborne exposure to potentially toxic cleaning agents often occurs, has been associated with a number of major birth abnormalities [221]. Gestational exposure to hormone disrupting chemicals has resulted in male offspring of hairdressers and agricultural workers being more likely to exhibit genital malformations [222]. As more and more evidence emerges about toxicant exposures in the workplace and associated adverse pediatric outcomes, it is clearly in the interests of society and individuals to be aware of the risks associated with assorted occupational exposures and for adequate precautionary avoidance to be instituted through locale change or credible air purification and ventilation in the workplace prior to the onset of pregnancy.

#### 4.1.3. Food Choices: Insecticide and Herbicide Exposures

There is escalating concern about the potential impact of various common pesticides that are in widespread use [149, 179, 223]. While some uncertainty remains about the definitive impact of all the available pesticides in common usage, repeated studies have shown that detectable exposure to assorted insecticides and herbicides is linked to neurological and cardiovascular problems, as well as increased rates of various cancers in offspring of exposed women [178, 180, 181, 224–230]. As a result, the discussion about the need to consume a credible organic diet remains a hot topic of debate.

While there has been disagreement about whether the nutritional content of credible organic food (where pesticide use has not been incorporated into the production of such food) is superior to regular conventional foods, the substantive difference is the higher amount of chemical residue found on conventionally grown produce (where pesticide use is employed) [231]. Various studies have confirmed that an organic food diet is associated with a significant decrease in the detectable levels of various toxic pesticides [231–233] and a consequent avoidance of maternal exposure to these agents. It might therefore be prudent to minimize intake of potentially toxic pesticides prior to and during gestation by consuming a diet low in pesticide residues.

#### 4.1.4. Exposure to Toxic Elements and Prenatal Supplement Contamination

It is increasingly evident that exposure to various toxic inorganic elements such as mercury, lead, and cadmium through contamination of foodstuffs [234], dental materials [235, 236], vehicle exhaust [237], and many other sources [235, 238, 239] is ubiquitous [240]. Cord blood studies have confirmed that developing children are routinely being exposed to toxic elements as a result of in utero contamination [72, 165, 241]. Accordingly, increasing study has endeavored to determine the potential toxicity associated with toxic metal and metalloid exposure. For example, gestational mercury exposure has received significant attention, as elevated maternal levels have been linked in offspring to impaired cognition, small for gestation age, and cardiovascular effects in later life [195, 196, 242–245]. Such findings have persuaded regulatory bodies to caution women against excessive consumption of contaminated seafood, a common source of toxic mercury exposure [79].

At low doses, gestational lead exposure has been associated with infant neurotoxicity and delayed development [193, 246, 247], as well as an increased risk of pediatric allergic disease [21]. Accordingly, over the last 50 years the defined “safe” level of lead exposure in pregnancy and childhood has been repeatedly revised and lowered as research uncovers the effects of increasingly minute amounts of this toxic heavy metal. More recently, it has been determined that no level of lead exposure appears to be “safe” and even what is defined as a “low” level of exposure in children may be associated with neurodevelopmental deficits [247]. The American College of Obstetricians and Gynecologists now recommends that all pregnant women be screened for lead exposure and, recognizing the serious risk, advises avoidance of breastfeeding if a certain measurable threshold is reached [248]. Fetal gestational exposure to other toxic elements including cadmium, arsenic, and titanium are also associated with adverse outcomes [249–255].

There are various potential and unsuspected sources of toxic element exposure. A couple of recent research studies, for example, have found various teas to be a source of toxic elements [234, 256]. Another noteworthy potential source of toxic element contamination for pregnant women is polluted prenatal supplements. It is routine for most women in the western world to consume prenatal supplements during pregnancy, the most common of which is a general vitamin and mineral supplement. Pregnant women are advised to do so in order to secure the nutritional needs of their developing child. However, a recent study confirmed that many prenatal supplements are contaminated with toxic elements, particularly lead, a bioaccumulative and teratogenic toxic element [71]. The daily ingestion of bioaccumulative toxic metals throughout pregnancy can present a serious danger and potentially have an adverse impact on the development and enduring well-being of the child.

Accordingly, it is important for maternity practitioners to caution their patients about this potential source of contamination and for such health providers to secure knowledge of safe, nonpolluted supplements. In addition, regulatory bodies may wish to consider published recommendations to increase the safety of commonly used supplements [71]. It may also be important to identify and label the source of supplement ingredients, as inexpensive raw materials originating from heavily polluted parts of the world are commonly used in manufacturing supplements and may be associated with more contamination [71].

In review, chemical toxicant exposure has become an expanding and ubiquitous problem. From morning to night, unsuspecting women are being exposed to a spectrum of toxic chemicals in their work, homes, community gathering sites, and places of leisure that have potential adverse impact on developing children. As discussed in paper entitled Nowhere to Hide [257], published nearly 10 years ago in Reproductive Toxicology: “Contemporary reproductive aged women and their offspring are facing an unprecedented onslaught of toxicant exposures from myriad sources in their day-to-day life” [257]. Matters relating to toxicology, however, have historically failed as a field to elicit efficient and timely decision-making in public health [258]. With extensive evidence now linking maternal toxicant exposures to adverse fetal outcomes, it is certainly time to respond.

### 4.2. Electrical Exposures

Another exposure that remains generally unrecognized in gestational care is electromagnetic radiation (EMR) [259]. Recent research is raising concern about the impact of exposure in pregnancy to energy fields emitted by wireless systems, power lines, various electronic devices, and ubiquitous mobile and cell phones [259–271]. Recent laboratory research from Yale University, for example, demonstrated that pregnant mice exposed to cell phone radiation produced offspring with hyperactivity and poorer memories compared to a nonexposed control group [263]. In human study, UCLA researchers studied large groups of mothers and children, finding that regular prenatal cellphone exposure to expectant mothers was associated with elevated risk for pediatric behavioral disorders and hyperactivity among their offspring [261, 262].

Recent evidence in human study also demonstrates that maternal EMR exposure emitted by mobile phones may lead to an increased fetal heart rate and decreased cardiac output [272] while close residential proximity to sources of extremely low frequency EMR in pregnancy is associated with a significant decrease in birth weight [273]. Furthermore, EMR exposure in pregnancy has been linked to a dose-dependent increase in the risk of miscarriage [274, 275]. Recent epidemiological evidence also suggests that maternal EMR exposure may also be linked to the development of asthma in offspring [276]. In addition, exposure to wireless radiation in general has the potential to damage or destroy neurological cells [277], and rats prenatally exposed to wireless radiation also show evidence of spinal cord damage [271]. While definitive conclusive evidence is hard to obtain as observational research may be confounded by a multiplicity of exposures, emerging evidence of potential risks associated with EMR exposure in pregnancy warrants a precautionary gestational approach. While some naysayers contend that such exposure is everywhere and cannot be avoided, simple measures can be taught through preconception and prenatal education to considerably diminish maternal and fetal exposure to EMR.

### 4.3. Emerging Exposures of Concern

#### 4.3.1. Paternal Exposures

Another area of consideration is the role of paternal exposure and birth outcomes. While not extensively studied, there are several indications that male chemical and electrical exposures can impact the developing child [216, 278]. For example, paternal exposure to certain solvents in the year prior to conception is linked with an increased rate of childhood cancer [279]. Specific occupations have been repeatedly linked to birth defects, including artists, landscapers and groundskeepers, gas and petroleum workers, sawmill employees, chemical workers, farmers, firemen, and printers [280–283], likely due to repeated job-related chemical exposures.

The exact mechanisms of toxicant-induced harm from the paternal side have not been elucidated but likely occur as a result of some combination of exposures. First, it may be that the father transfers toxicants to his partner in semen and thus elevates her toxicant burden. Secondly, the paternal exposure may affect the genetics of his sperm, which eventually fertilize the egg and thus influence fetal development [284]. Finally, the father may come home with contamination from his work on clothing that the mother may inhale on contact or through washing clothes. It is important that practitioners alert couples to the possibility of such exposure.

In addition to adverse chemical exposures, there is also concern about the fetal impact of paternal EMR radiation. With an increased tendency towards malformations among children of men in some high EMR exposed occupations [285], researchers have surmised that paternal EMR exposure may be a factor in adverse gestational and pediatric outcomes. Significant electrical exposure of fathers, for example, has been associated with higher rates of preterm birth [286]. The developments of atypical sperm, chromosomal aberrations, and congenital defects in offspring have also been linked in some cases to male EMR exposure [285, 287–290]. Fathers employed in industries with higher than average EMR exposure have been noted to have offspring with higher rates of subsequent brain and spinal cord tumours [287, 288]. More research needs to be undertaken to conclusively determine the link between paternal EMR exposure and reproductive outcomes, but preliminary evidence suggests that young couples should be apprised of the potential risk so that precautionary measures may be considered.

#### 4.3.2. Nanoparticles

There continues to be the manufacture and release of new types of potential toxicants for which there are uncertain sequelae on human health. Expanding production and release of engineered nanoparticles, for example, may be affecting human health including the health of pregnant mothers and their offspring [291]. In developed cultures, these ultraminuscule agents are being incorporated into an ever-increasing number of products. For example, reproductive aged women are routinely exposed through some processed foods containing nanoparticles [292] (increasingly being used as a mechanism for flavor enhancement) and through cosmetics for esthetic purposes [293] as well as in sunscreen lotions.

The field of nanotoxicology [294] is gathering more and more attention with the finding that these exceptionally small agents can bypass human defense mechanisms and penetrate into cells and disrupt biological function [295–297]. Of particular note is emerging animal study that raises concern in relation to gestational exposure to some types of nanoparticles [298]. Recent study, for example, confirms that nanoparticles cross the placenta, some nanoparticles are linked to fetal neurotoxicity [299], and specific nanoparticles are associated with structural and functional abnormalities within the placenta [299]. Simple instruction can be provided to prospective mothers alerting them to the risks of nanoparticle exposure and educating them about means to minimize exposure to these potentially teratogenic agents.

#### 4.3.3. Genetically Modified Foods

There has been widespread manufacture, release, and consumption of genetically engineered or transgenic foods—foodstuffs where the basic genetic material of the food has been modified to allegedly enhance some aspect in the continuum of food growth, production, and food provision. For example, there has been the recent manufacture and release of genetically modified salmon; in this case, the gene modification results in persistent release of growth hormone to facilitate enlarged fish to purportedly increase food availability [300]. While unchecked growth hormone in humans is considered pathological, long-term outcomes of consuming fish tissues stimulated by incessant growth hormone are uncertain. There has also been concern expressed about the potential impact of uptake and incorporation of modified genetic material from such foods into the human biome [301], as recent study has confirmed that components of genetically modified foods are now found in nearly all pregnant women and their developing children [302].

Much is uncertain about the potential risks and consequences associated with the consumption of transgenic foods as this recent technology does not have the benefit of independent long-term outcome study. As many processed foods now contain genetically modified components, however, this issue has become an intensely controversial topic, particularly as there is no regulation to label such foods and to provide consumers with choice in food selection. While many food regulators in North America contend that these ingested products are totally safe, regulators in some other jurisdictions have come to different conclusions resulting in the banning of such foodstuffs in many European nations. Until more is known about the lasting impact of these food alterations, some maternity health providers have encouraged and taught a precautionary approach in order to preclude any yet unforeseen risk.

## 5. The Economic, Emotional, and Social Costs of Preventable Gestational Complications

The short- and long-term fiscal expenditures associated with preventable gestational complications are enormous and provide an incalculable load on already overburdened healthcare systems. According to FIGO, the global health and economic burden related to toxic environmental chemicals is in excess of billions of dollars every year [14]. The enduring costs of caring for offspring with chronic disabilities as a result of health problems resulting from gestational toxic exposure are untold. Nutritional deficiency in pregnancy is also associated with massive consumption of resources [76]. For example, preterm birth appears to be a common consequence of nutrient deficiency [76, 124, 126]. As mentioned, the annual costs associated with premature births are overwhelming [6]. Furthermore, many health problems sustained by children born prematurely continue far beyond their childhood years [5] with ongoing economic encumbrance placed on health, education, and social service resources.

The emotional labour and cost to families caring for children with chronic illness are also enormous [303–305]. A study of mothers whose infants were diagnosed with health problems soon after birth found these women to be at a significantly greater risk of developing postpartum depression [306]. If the childhood diagnosis is not fatal, the ongoing care rarely ends during the pediatric years as the family will usually have to contend with additional, long-term healthcare needs and/or special educational services to optimize their offspring's health outcomes. While these efforts are valiant, prevention of enduring suffering and hardship for families is clearly preferable.

The issue of autism spectrum disorder (ASD) in America provides a clear example which highlights the social costs of preventable gestational complication and the need for urgent action in the area of reproductive health services. Autism was a relatively uncommon condition with a prevalence rate of about 2–5 per 10,000 in the 1950s [307]. The occurrence of this chronic neuropsychiatric condition rose steadily to about 1 in 2500 in the mid-1980s and to 1 in 150 by 2002 [308]. The incidence of ASD continued its colossal ascent to a 2015 estimated prevalence by the Centres for Disease Control of 1 in 45 or 2.24% of the pediatric population [309], with no sign of abatement in the near future. Furthermore, there are some local pockets in America such as the Somali community in Minnesota with a 2008 reported rate of 1 in 28 [310]. This is of particular interest since this community often refers to autism in children as the “American Disease” or the “Minnesota Disease” since most in the community report never having heard of it among children in their country of origin. Based on recent and current trends, some research scientists anticipate ASD prevalence rates that are unthinkable over the next two short decades [311]. While genomic research suggests that genetic predisposition can be identified in a smaller percentage of ASD children, mounting evidence submits that exposure of the fetus or infant to adverse environmental toxicity is a significant determinant of this condition [15].

The personal, fiscal, and social costs of this disorder are enormous indeed. As well as the enduring challenges faced by families and healthcare systems in caring for these precious children, the emerging concern about provision and care for the rapidly swelling numbers of young and soon-to-be aging adults with this mental disability is just beginning to unfold. Many parents of ASD children are fearfully asking where the resources will come from and who will provide care for their children when parents are aged and pass on. With the continuing rise of disabled people unable to take care of themselves, to earn a living, or to contribute to the tax base as a result of the rapidly escalating pandemic of chronic illness [312], there is concern about a growing caretaker society where there are more people requiring care than there are people able to take care of them. Preventing ASD and other developmental afflictions by addressing modifiable gestational determinants that are often etiologically involved in such disorders has evident benefits.

## 6. Practical Application of Preconception Care

The medical community has made great efforts to develop and deliver prenatal care over the last few decades, resulting in a significant decline in the number of women who do not receive gestational healthcare, and with overall improved outcomes for at-risk populations [313, 314]. At this point in history, however, a leading maternal health journal has declared that the next “maternal and child health frontier of prevention” lies in preconception care (PCC) [315]. Furthermore, the March of Dimes, an international advocate for maternal and child health, agrees that primary care providers should be taking every opportunity to provide this kind of care for reproductive aged women at “the time when it really can make a difference” [316].

In recent study as to why health providers have failed to provide comprehensive information about toxic exposures in pregnancy despite abundant information in the scientific literature, some respondents claimed that they worried about inflicting stress on pregnant women [317]. Although this is a legitimate concern in the immediate situation, the long-term sequelae of failing to provide crucial instruction in order to preclude adverse gestational outcomes have the potential to induce much more enduring stress and difficulty for all involved. It is important to be aware that rather than taking a negative and fear-inducing approach with patients, it is preferable and more effective from the authors' considerable experience in this area to educate patients on how to “creatively engage” with the realities of the modern world in their particular circumstances. Rather than general platitudes about diet and exposure avoidance, individual patients and couples need to learn how to meet their specific needs.

Accordingly, there has been more and more call for a multifaceted approach to PCC (preconception care) [14, 315]: (i) public education; (ii) government regulation of toxicant release into the environment; (iii) provision of targeted group instruction; and (iv) provision of personalized services to address particular requirements. In some sense, all women of reproductive age are potentially preconceptive, and public health measures to educate them about gestational requirements as well as exposures and avoidance could make a substantial difference in outcomes. Although public and societal services and government regulation are beyond the scope of this paper, consideration of healthcare services by maternity health providers will be discussed.

The preconception period is of particular importance for several reasons:

- Most women do not begin to receive prenatal care until part way through or near the end of their first trimester. At this point, much of the critical development has already occurred as the fetus has formed the beginnings of all of its major organs. The first trimester, therefore, represents a most crucial time of exposure avoidance and adequate nutrition.
- Given that some women feel fatigued or less than optimal during the first trimester of pregnancy, it is a challenging time to start suggesting that they make significant lifestyle changes. If they are apprised of necessary information prior to pregnancy, their ability to plan and prepare is greatly increased.
- Certain interventions are not advisable during pregnancy (i.e., specific immunizations, detoxification); and appropriate interventions performed prior to conception can optimize outcomes.
- Certain medical conditions and pharmaceutical use are best addressed ahead of conception (i.e., diabetes control, antidepressant medication [318]) and may require significant amounts of time to effectively address (i.e., smoking cessation, alcohol/drug use).

Accordingly we recommend and offer PCC to all reproductive aged women in the form of generalized instruction as well as individualized personal care.

Generalized classes are offered in our clinic which provide an overview of important gestational determinants discussed in the scientific literature and which offer lifestyle and environmental choices that deliver the best chance of having optimal pregnancies and delivering healthy, happy children. Instructions on practical aspects of nutrition, dietary measures, toxicant avoidance, prudent supplement use, and other lifestyle interventions are systematically discussed (see Appendix). Measures to secure clean air, water, and foodstuffs are incorporated into group discussions. These are provided on a regular basis at the medical clinic and are well-received.

Personalized PCC services are offered by physicians in our clinic. Dietary and nutritional histories are taken and environmental exposure inventories are completed (see Appendix) and discussed in detail. Required counselling to provide practical solutions to preclude toxic exposures is included in the preconception care visits. Patients should be educated about how to avoid toxic environmental chemicals and providers should learn about resources in the community that can assist in education. In our view, all members of the reproductive healthcare team need to be apprised of the information found in the scientific literature and to acquire the necessary skills to provide this type of instruction.

For patients keen to be assessed for an already existing internal dose of toxicant bioaccumulation, toxicological testing is discussed and offered. If individuals are found to have accrued a considerable dose of toxicants on testing, medical intervention is provided to address and substantially eliminate the burden of toxicants prior to pregnancy [206, 207, 209, 210, 319, 320] in order to preclude exposure to the fetus. Eliminating the toxicant burden and precluding the passage of teratogens to the vulnerable fetus have clear benefit.

*Examples of Specific Recommendations Routinely Discussed in Preconception Care*

*(I) Secure Food Health and Safety *

 Caution must be taken with supplements chosen during gestation [71].
 Minimize refined sugar intake in pregnancy [321].

*(II) Secure Maternal and Fetal Sufficiency *

 Avoid common nutritional deficiencies in pregnancy (Table 1).
 Secure optimal fetal biome development:
   For example, consider vaginal swab seeding in Caesarean sections [161].
   For example, avoid gestational antibiotic use if possible.

*(III) Precautionary Avoidance of Adverse Chemical Exposure [14]*

 Avoid exposure to adverse chemicals in air, water, and food, as much as possible [49].
 Avoid foods that are contaminated with pesticides [322].
 Avoid seafood in pregnancy [79].

*(IV) Precautionary Avoidance of Electromagnetic Radiation [259–271] *

 Avoid carrying mobile phones, cordless or cell phones, anywhere on the body.
 Only use cell phones on a speaker setting as far away from the body as possible.
 Avoid the use of wireless systems in the home or car.
 Establish a hard-wired system for computer use in the home and workplace.
 Avoid being in close proximity to routers and smart meters.

## 7. Conclusion

It now appears that while hazards of gestation to the mother have receded considerably over the last many years, hazards to the fetus seem to be growing. Moreover, while increasing attention has been devoted over the last decade to screening techniques in order to identify fetal abnormalities and to provide the option of pregnancy termination, there is little recognition that many obstetrical and fetal health problems can be entirely precluded if adequate precaution is taken. Modification of such determinants has the potential to prevent the development of maternal or fetal problems and also to obviate the difficult choice of pregnancy dissolution. Furthermore, the expanding range of fetal screening measures is unable to predetermine the overwhelming majority of common pediatric afflictions such as autism, allergic disease, pediatric cancer, and learning disability. The recent realization that many of these conditions are often related to modifiable gestational determinants [15, 31, 76, 134, 185] and can be prevented in many cases by informed preconception and gestational choices demands an official response from the medical community. So what can be done?

Nelson Mandela's admonition that “education is the most powerful weapon which you can use to change the world” is particularly apposite with regard to preconception care. Although there are some individuals who, despite adequate education, will continue to engage in high risk activities for fetal harm in pregnancy such as smoking and alcohol use, most prospective parents are keen to be apprised of and to implement necessary measures to maximize health outcomes for their developing child. With the majority of the gestational population deficient in required nutrients [76] and recent cord blood testing confirming widespread prepollution with disease-inducing toxic chemicals [72, 165], there is much that remains to be done to optimize outcomes in pregnancy. It appears unethical and unscientific for the medical community to withhold essential and available information from prospective parents which would empower them to avoid serious preventable illness in their children. Furthermore, with the totality of available scientific evidence that now exists in the literature on the potential to modify disease-causing gestational determinants, failure to implement necessary precautionary education may render members of the medical community collectively and individually culpable and liable for preventable illness in children. So where does the problem rest?

A recent survey of American obstetricians revealed that the overwhelming majority of maternal health providers have come to recognize the profound impact of environmental determinants on reproductive health and the instrumental role that physicians might play in prevention of obstetric and pediatric sequelae [317]. Despite this awareness, however, less than 20% of respondents routinely question their patients about potential adverse exposures [317], as more than 90% claim they have had no training in the field of environmental health sciences and toxic exposures [317]. While most physicians have the skills to provide education about smoking, alcohol, and drug cessation, the plethora of other equally or more serious potential exposures continues to accumulate and health providers lack training in assessing or dealing with this modern reality. Furthermore, broad training about practical nutritional biochemistry including requirements and common deficiencies has long been absent in the education of medical health professionals [323].

The special communication by FIGO at the recent scientific assembly recommended that environmental health becomes a fundamental part of healthcare [14] and concluded that “on the basis of accumulating robust evidence of exposures and adverse health impacts related to toxic environmental chemicals, the International Federation of Gynecology and Obstetrics (FIGO) joins other leading reproductive health professional societies in calling for timely action to prevent harm” [14]. Along with societal efforts through educational systems, government programs, and media initiatives to secure knowledge translation in the area of preconception care, it is the authors' recommendation that such care with explicit education and instruction about common deficiencies as well as avoiding and addressing toxicant exposure and bioaccumulation be adopted immediately and become the required standard of care in reproductive health services; the consequences for individuals, for society, and for the medical community of failing to do so are far too high.

## Figures and Tables

**Table 1:** Common deficiencies in pregnancy.

Deficiency	Associated effects	Treatment
Vitamin D deficiency	Myriad potential sequelae (see Table 2), for example, increased risk for decreased bone mass, respiratory issues, short-sightedness, and crooked teeth in offspring	Assessment of maternal serum levels in preconception period and supplementation as necessary-seasonal adjustments may be required [78]

Docosahexaenoic acid (DHA) deficiency	Preterm labour and postpartum depression in mothers Central nervous system disturbance, poor sleep pattern, lower IQ, and impaired blood pressure control later in life in offspring	Distilled cod liver oil supplement prior to and throughout pregnancy [79]

Magnesium deficiency	Potential determinant of adverse gestational outcomes [80, 81]	Supplementation in preconception period Education about foods high in magnesium

Compromised microbiome environment	Preterm labour [82] Serious bacterial infection in first 3 months of life in offspring [83] and potential long-term pediatric complications [84]	Avoidance of unnecessary antibiotics Good quality probiotic Fermented foods and drink in preconception and prenatal period

**Table 2:** Potential sequelae of maternal vitamin D deficiency in pregnancy.

Association	Specific study finding
*Maternal sequelae*	

Early pregnancy loss	47% of women with repeated pregnancy loss found to have vitamin D deficiency [85]
	Vitamin D deficiency associated with twofold increased risk of first trimester miscarriage [86]

Gestational diabetes	Third trimester serum 25(OH)D level inversely correlated with HbA1c [87]
	Significantly lower serum 25(OH)D levels found in women with glucose intolerance and GDM at 24–28 weeks of gestation [88]

Preeclampsia	In nulliparous women, 25(OH)D levels were 15% lower in early pregnancy for those who went on to develop preeclampsia compared to those who did not; women with serum level < 37.5 nmol/L had 5-fold increased odds of developing preeclampsia [89]
	Women with preeclampsia had significantly lower vitamin D levels in mid-late pregnancy [90]
	Maternal and umbilical cord serum 1,25(OH)2D levels were significantly lower in women with preeclampsia as compared to controls [91]

Preterm births	Incidence of preterm birth increased significantly as serum 25(OH)D levels decreased [92]
	Women with preterm births before 31 weeks had nearly double the rate of vitamin D deficiency as controls [93]

Higher rates of Caesarean section	Women with a 25(OH)D level less than 37.5 nmol/L had nearly quadruple the rate of Caesarean sections than those with levels greater than 37.5 nmol/L [94]

Small for dates infants	Women with vitamin D deficiency had a 12 times increased relative risk for low birth weight babies compared to controls with sufficient vitamin D [95]

Maternal postpartum depression	Women with 25(OH)D serum levels less than 35.4 nmol/L had a 7-fold increased risk of developing postpartum depression [96]

*Sequelae in offspring*	

Impaired lung development	Maternal vitamin D deficiency at 18 weeks associated with poorer lung function and increased risk of wheezing at age 6 [97]
	Lower maternal vitamin D intake in pregnancy associated with persistent wheeze in 5-year-old offspring [98]

Neurocognitive development	Maternal vitamin D < 70 nmol/L at 18 weeks gestation associated with nearly twofold increase in impaired language development at age 5 and 10 in offspring [99]

Bone strength	Maternal vitamin D < 50 nmol/L in midpregnancy associated with lower peak bone mass in offspring at 20 years of age [100]
	Maternal vitamin D deficiency in late pregnancy associated with reduced bone mineral content in offspring at age 9 [101]

Eating disorder	Maternal vitamin D deficiency at 18 weeks of pregnancy associated with 1.8-fold increased risk of development of adolescent eating disorder in offspring [102]

Multiple sclerosis	Lower maternal vitamin D intake in pregnancy associated with elevated risk of development of multiple sclerosis in offspring [33]

**Table 3:** Examples of gestational toxicant exposures and associated outcomes.

Toxicant	Sources of exposure	Study findings
Flame retardant chemicals	Polyurethane foam in mattresses, furniture, carpet padding, car seats, and so forth (particularly those made before 2005)	Cord blood flame retardant levels linked to impaired mental and physical function at 1, 3, and 6 years of age [16]
		Prenatal PBDE exposure linked to decreased IQ and increased hyperactivity at 5 years of age [169]
		Dose-dependent inverse relationship between serum levels of PBDEs and thyroid stimulating hormone in pregnant women [170]

Bisphenol A	Hard plastics used in food storage, leaching from lining of canned food and drink, water coolers, dental sealants, contact lenses	Prenatal exposure to bisphenol A associated with persistent wheezing in offspring [171]
		Elevated maternal serum BPA at delivery associated with increased risk of low birth weight babies [172]
		Twofold increase in first trimester maternal serum BPA associated with 55 g less birth weight in offspring [173]

Phthalates	Plasticizer in soft plastics, fragrances, perfume, cosmetics, paint, flooring	Prenatal phthalate exposure linked to 70% increased risk of asthma [174]
		Maternal phthalate levels linked to decreased IQ in offspring at 7 years of age [175]
		Elevated phthalate metabolite urine concentration associated with increased risk of spontaneous abortion in dose-dependent fashion [176]
		Elevated phthalate metabolites in maternal urine in early pregnancy associated with decreased anogenital distance in male offspring [177]

Pesticides, insecticides, herbicides	Nearby farms, parks, cemeteries, golf courses; pesticide residue on foods Spraying on lawns of patient and neighbours	Pregnant women living within 1.5 miles of an area sprayed with pesticides and insecticides associated with 60% increase in autism spectrum disorder [178]
		Agricultural pesticide exposure associated with 2-fold increase in odds of developing gestational diabetes [179]
		Every standard deviation increase in chlorpyrifos (a common insecticide) exposure corresponded to 1.4% decline in IQ and 2.8% decline in working memory in 7-year-old children [180]
		In a meta-analysis, OR was 2.1–2.4 for childhood leukemia with prenatal maternal occupational pesticide exposure; risk also elevated with prenatal maternal occupational exposure to insecticides (OR 2.72) or herbicides (OR 3.62) [181]

Solvents	Occupational exposures (cleaners, nurses, hairdressers, chemists) Inhalational exposure from paints, cleaning products, cosmetics, air pollution from nearby industry	Parental exposure to solvents associated with a nearly 3-fold increased risk of autism spectrum disorder in offspring [182]
		Solvent exposure in early pregnancy associated with dose-dependent increased risk of birth defects, particularly oral clefts, urinary tract malformations, and male genital malformations [183]
		Occupational exposure to solvents in first trimester of pregnancy associated with 13-fold increased risk of major malformations [28]
		From 3-month preconception through to the end of breast feeding period, parents of children with autism are more likely to have been exposed to lacquer, varnish, xylene, asphalt, and other solvents compared to parents of controls [184]

Air pollution	Benzene and other volatile gases from car exhaust, petroleum derived volatile chemicals from nearby industry, (oil refineries, car factories/repair shops), and so forth	Childhood cancers geographically associated with birth address of mother when proximate to specific industries and airborne exposures [31, 185]
		Exposure to ozone and fine particulate matter associated with increased risk of gestational hypertension and preterm delivery [186]
		Increased risk of low birth weight and premature infants with increasing exposure to sulfur dioxide and measured levels of total suspended particles [187]
		25–51% increased rate of Hodgkin's lymphoma in offspring with maternal high exposure to traffic-related air pollution in pregnancy [188]
		Maternal residence in pregnancy proximate to a freeway more common in mothers of children with autism than mothers of controls [189]

Home renovation	VOCs from flooring and painting; flame retardants in furniture and carpets; formaldehyde from particleboard; and so forth	Home renovation or redecoration within past 12 months (including flooring, painting, and new furniture) associated with increased risk of wheezing, allergy, and asthma symptoms in offspring [190] New furniture in the home in the year before birth associated with significant increase in wheezing, allergic rhinitis, and eczema in offspring [191]

Heavy metals	*Lead*: old, flaking lead paint, cosmetics, food contamination from elevated soil levels, old water pipes, and so forth	Maternal exposure to levels of lead as low as 5 *μ*g/dL associated with lower developmental functioning in newborns, particularly if exposed in first trimester [192]
		Maternal lead exposure related to significant decrease in Mental Development Index [193]
	*Mercury*: dental amalgams, dietary consumption (seafood), and so forth	Significant relationship between fish consumption in pregnancy and mercury levels in mothers and newborn infants [194]
		6- and 7-year olds scholastic and psychological test scores significantly associated with mercury levels in mothers during their pregnancy [195]
		7-year-old children neurophysiological testing demonstrated association between elevated maternal mercury level in pregnancy and lower testing scores [196]
		For every 1000 lb of environmentally released mercury, a corresponding geographical 61% increase in autism rates was found [197]

## Appendix

The following is an extract from Preconception Care Questionnaire for patients.

### Preconception Care Questionnaire

Name: —

Pregnancy is a very exciting time in a woman's life. It has the potential to bring great joy and anticipation as you await the arrival of your child. When challenges arise in pregnancy or with the developing child, families can suffer significant anxiety and uncertainty. By creatively engaging with some of the realities of modern society, decisions and choices can be made to optimize the outcomes of your pregnancy and the health and well-being of both mother and child(ren). In this Preconception Care (PCC) program, every effort will be made to provide you with practical information that will allow you to maximize your health and the health of your future child(ren). To clarify, PCC (preconception care) is the care of women* prior to their conceiving a child*, in order to optimize their health during pregnancy and the health of their future children.

The International Federation of Gynecology and Obstetrics, a medical group which monitors maternal health care throughout the world, recently released a special communication regarding a particular concern for pregnant women. They stated that “*Exposure to toxic environmental chemicals during pregnancy and breastfeeding is ubiquitous and is a threat to healthy human reproduction. There are tens of thousands of chemicals in global commerce, and even small exposures to toxic chemicals during pregnancy can trigger adverse health consequences*”. In our clinic, we endeavor to make sure you will receive all the nutrients you need in pregnancy, but also carefully assess for possible exposures that might cause problems for you or your developing child(ren). By learning about measures to avoid or eliminate toxic exposures, you can avoid the risks and worry associated with adverse health consequences as mentioned above.

With PCC, we aim to educate patients and their partners about the potential benefits and harms in their everyday environment and to teach them how lifestyle modifications can have a profound and positive impact on health outcomes for them and their children. This program will approach patient care by regularly reviewing emerging scientific literature and translating that information into practical recommendations for healthy living and optimal health.

Through a series of individual appointments, you will be invited to review the questionnaire with the physician. There are also biweekly classes at our clinic. There are specific classes which are particularly relevant to this program that you will be encouraged to attend, but an entire class schedule will be available to you.

We look forward to working with you,

Dr. Stephen J. Genuis and Dr. Rebecca A. Genuis.

*Diet and Nutrition*

Please give three average (for you) daily breakfasts:
  —
Please give three average (for you) daily mid-morning snacks:
  —
Please give three average (for you) daily lunches:
  —
Please give three average (for you) daily mid-afternoon snacks:
  —
Please give three average (for you) daily dinners:
  —
Please give three average (for you) daily evening snacks:
  —
Which supplements do you take?
  —
Do you eat seafood?
  No—
  Yes—
  How many servings per week? —
  Type of seafood (e.g., salmon, tuna, etc.) —

What percent of your diet is organic?
  Regular—%
  Organic—%
What percentage of the food you consume is
  Cooked? —%
  Raw? —%
Do you eat genetically modified foods?
  No—
  Yes—
  Don't know—
Do you eat irradiated food?
  No—
  Yes—
  Don't know—
Do you eat food with trans-fat?
  No—
  Yes—
  Don't know—
Are you exposed to fumes from cooking oils?
  None—
  Little—
  Moderate amount—
  Quite a lot—
What kind of oil do you use to cook with?
  —
Do you eat a lot of processed food?
  Never—
  Rarely—
  Moderate Amount—
  Frequently—
Do you use artificial sweeteners such as Aspartame, Splenda or Nutra Sweet?
  None—
  Little—
  Moderate amount—
  Quite a lot—
On average, how many times per week do you eat at restaurants or fast food establishments?
  —

Do you have an induction stove top?
  No—
  Yes—
Do you eat margarine regularly?
  No—
  Yes—
Do you use aluminum cookware?
  No—
  Yes—
Do you use any non-stick cookware?
  No—
  Yes—
How much of the following beverages do you consume regularly and have you linked any symptoms with drinking them?
Beer: Number of bottles per week—
Wine: Number of glasses per week—
Coffee - Caffeinated: Number of cups per 24 hours—
Coffee - Decaffeinated: Number of cups per 24 hours—
Regular Tea: Number of cups per 24 hours—
Herbal Tea: Number of cups per 24 hours—
Pop - Regular: Number of glasses per 24 hours—
Diet Pop: Number of glasses per 24 hours—
Do you regularly drink from plastic containers?
  No—
  Yes—
Do you drink carbonated (fizzy) drinks?
  Never—
  Rarely—
  Moderate Amount—
  Frequently—
Do you eat fruits and vegetables on a regular basis?
  No—
  Yes—
  How many servings/day?—
Do you eat raw nuts?
  No—
  Yes—
  How many servings/week?—

*Human Chemical Exposure Assessment*

*(1) Exposure to Smoke*

Are you currently a smoker?
  No—
  Yes—
If yes, average number of cigarettes per day:
  —
If yes, total number of years smoked throughout your life?
  — years
If yes, how many times have you previously tried to quit smoking?
  —
If not currently smoking, have you ever been a smoker?
  No—
  Yes—
If yes, for how many years?
  —
If stopped smoking previously, which year did you stop?
  —
Are you the regular recipient of second-hand smoke?
  No—
  Yes—
Are you often exposed to smoke from candles or campfires?
  No—
  Yes—
Are you often exposed to smoke from barbeques?
  No—
  Yes—

*(2) Inhalant Exposures*

Are you regularly exposed to:

Fabric Softener
  No—
  Yes—

Scented Products
  No—
  Yes—
Glues
  No—
  Yes—
Bleach
  No—
  Yes—
Perfume/Cologne
  No—
  Yes—
Incense
  No—
  Yes—
Furniture Polish
  No—
  Yes—
Disinfectants
  No—
  Yes—
Air Fresheners
  No—
  Yes—
Printers/photocopy machines?
  No—
  Yes—
Gasoline fumes
  No—
  Yes—
Paint thinner
  No—
  Yes—
Nail Polish/Remover
  No—
  Yes—
New carpet
  No—
  Yes—

Paint
  No—
  Yes—
Dry cleaning
  No—
  Yes—
Cosmetics
  No—
  Yes—
Solvents
  No—
  Yes—
Industry fumes
  No—
  Yes—
Dust
  No—
  Yes—
Do you have Hepa filtration in your home?
  No—
  Yes—
  Please provide details—
Do you have a mechanism to have ongoing ventilation in your home?
  No—
  Yes—
  Please provide details—

*(3) Mold & Mycotoxins*

Is there mold in your home or workplace?
  Yes—
  No—
  Don't know—
Have you ever been exposed to indoor mold?
  Yes—
  No—
  Don't know—
Do you have a humidifier in your home?
  Yes—
  No—

Do you have any water stains on your ceilings?
  Yes—
  No—

*(4) Motor Vehicles*

How much time do you spend in total in the average day in a motor vehicle?
  —
Has your car ever had treatment for seat, leather, upholstery or stain protection?
  No—
  Yes—
  Don't know—

*Human Electrical Exposure Assessment*

Are you aware of the health concerns related to electromagnetic radiation exposure?
  Aware—
  Have heard of it—
  Not aware—
Do you use a cell phone or tablet on a regular basis?
  No—
  Yes—
Do you use a cordless phone on a regular basis?
  No—
  Yes—
Do you use compact fluorescent light-bulbs in your home?
  No—
  Yes—
Do you have any electric heating in your home?
  No—
  Yes—
Do you use an electric blanket?
  No—
  Yes—
Do you use a heating pad?
  No—
  Yes—

Do you spend much time under fluorescent lights?
  No—
  Yes—
Do you have any dimmer switches in your home?
  No—
  Yes—
Do you use tanning salons?
  Never—
  Occasionally—
  Regularly—
Do you have an electrical device such as a clock radio near to your head overnight?
  No—
  Yes—
Do you currently live close to overhead Power Lines?
  No—
  Yes—
Do you currently live close to a Power Generating Station?
  No—
  Yes—
Are various power and electrical devices in close proximity to your body on a regular basis?
  Hair dryer
    No—
    Yes—
  Electric shaver
    No—
    Yes—
  Vacuum cleaner
    No—
    Yes—
  Power tools
    No—
    Yes—
  Massage Chair
    No—
    Yes—
  Sewing machine
    No—
    Yes—
Do you use an electric or battery powered toothbrush
  No—
  Yes—
Do you use a laptop computer on your lap?
  No—
  Yes—
  How often?—
Do you have a wireless computer network in your home or workplace?
  No—
  Yes—
  Home—
  Workplace—
  Both—
Do you have a wireless security system in your home or workplace?
  No—
  Yes—
  Home—
  Workplace—
  Both—
Do you have a communications satellite emitting signal close to your home or workplace?
  No—
  Yes—
  How close?—
Are you aware of the health concerns related to dirty electricity or stray voltage?
  Yes—
  No—
Does your car have a seat warmer that you sometimes use while driving?
  Yes—
  No—
  Don't know—
Does the electrical power enter your residence near your bedroom?
  Yes—
  No—
  Don't know—
Is there a power transformer in your yard?
  Yes—
  No—
  Don't know—
